# Toward Generating Subtype-Specific Mesencephalic Dopaminergic Neurons *in vitro*

**DOI:** 10.3389/fcell.2020.00443

**Published:** 2020-06-17

**Authors:** Tiago Cardoso, Martin Lévesque

**Affiliations:** ^1^Department of Psychiatry and Neurosciences, Faculty of Medicine, Université Laval, Québec, QC, Canada; ^2^CERVO Brain Research Center, Université Laval, Québec, QC, Canada

**Keywords:** Parkinson’s disease, cell replacement therapy, pluripotent stem cells, dopaminergic neurons, induced neurons, single cell sequencing, cell reprogramming

## Abstract

Mesencephalic dopaminergic (mDA) neurons derived from pluripotent stem cells (PSCs) have proven to be pivotal for disease modeling studies and as a source of transplantable tissue for regenerative therapies in Parkinson’s disease (PD). Current differentiation protocols can generate standardized and reproducible cell products of dopaminergic neurons that elicit the characteristic transcriptional profile of ventral midbrain. Nonetheless, dopamine neurons residing in the mesencephalon comprise distinct groups of cells within diffusely defined anatomical boundaries and with distinct functional, electrophysiological, and molecular properties. Here we review recent single cell sequencing studies that are shedding light on the neuronal heterogeneity within the mesencephalon and discuss how resolving the complex molecular profile of distinct sub-populations within this region could help refine patterning and quality control assessment of PSC-derived mDA neurons to subtype-specificity in vitro. In turn, such advances would have important impact in improving cell replacement therapy, disease mechanistic studies and drug screening in PD.

## Introduction

The role of mesenscephalic dopaminergic (mDA) neurons in cognition, motivation, motor control and its association with several neuropsychiatric disorders, in particular Parkinson’s disease (PD), has made this discrete cellular population the subject of extensive investigation for decades (Bjorklund and Dunnett, 2007b). mDA neurons share the common characteristic of producing and utilizing dopamine as their primary neurotransmitter and have been traditionally classified into three distinct regions, the retrorubral field (A8), the substantia nigra pars compacta (SNc; A9), and the ventral tegmental area (VTA; A10) (Bjorklund and Dunnett, 2007a). The classification of these neurons into distinct anatomical clusters has been primarily been based on their location, cellular morphology, molecular profile, electrophysiological properties, and area of projection (Roeper, 2013; Anderegg et al., 2015). However, the complexity of behaviors associated to mDA neurons and their different vulnerability profile cannot be fully explained by this conventional classification. Recent efforts on single cell mRNA sequencing are now starting to shed a light on the complex heterogeneity of adult mDA neurons, by describing molecular identifiers of different populations of mDA neurons and providing updated classification of neuronal clusters (Poulin et al., 2014; La Manno et al., 2016; Tiklová et al., 2019). This knowledge now permits for a tailored interrogation of the connectivity, physiological characteristics, and functionality of the different subtypes of mDA neurons (Poulin et al., 2018).

Pluripotent stem cells (PSCs) have the unique capacity of self-renewal and the potential to become any cell type in the body. It is not surprising then, considering the multitude of applications ranging from cell therapy, disease modeling, and drug screening, that tremendous efforts to generate *bona-fide* mDA neurons have been undertaken (Arenas et al., 2015), with particular interest in PD. Indeed, it is now possible to generate stem cell-derived mDA neurons that express canonical midbrain markers such as Lmx1a, and FoxA2, and that can be transplanted into animal models of PD to survive, release dopamine, integrate into host circuitry, and function as proper mDA neurons (Kriks et al., 2011; Kirkeby et al., 2012, 2017; Grealish et al., 2014; Kikuchi et al., 2017; Niclis et al., 2017; Cardoso et al., 2018; Adler et al., 2019). However, despite the incredible advancements in the field and the capacity to obtain high percentages of mDA neurons in culture, it still remains a challenge to generate specific subtypes of mDA neurons. This is of particular importance if considering the different characteristics of mDA neuronal subtypes in terms of vulnerability, target of innervation, function in the brain, and involvement in different aspects of neuropsychiatric disorders. For instance, PD is characterized by the selective loss of mDA neurons of the SNc, with the consequent denervation of dorsal regions of the striatum causing motor symptoms associated to the disease (Dauer and Przedborski, 2003). It is therefore necessary to produce the appropriate subtype of mDA for the efficient application of cell therapy in PD (Grealish et al., 2010). Moreover, tailoring differentiation to specific subtypes would be invaluable for disease mechanistic and drug screening studies. There is therefore a need for a better comprehension on the molecular fingerprints that identify different subtypes of mDA neurons, and the key molecular regulators involved in their specification during development. This will allow for the optimization of differentiation protocols, and quality control assessment of the cell product composition, and fidelity.

In this review, we discuss how recent knowledge in mDA neuronal development and diversity stemming from single cell transcriptome profiling investigations may impact technological development toward generating induced mDA neuronal subtypes, with a particular focus on the potential application for PD research and therapy.

## Generating mDA Neurons in the Dish: Strategies, Applications and Perspectives for PD

PSCs are, by definition, self-renewable and hold the potential to differentiate into any somatic cell type of the body. While embryonic stem cells (ESCs) are derived from the inner mass of a blastocyst – a pre-implantation early stage embryo (Thomson et al., 1998) –, induced pluripotent stem cells (iPSCs) are derived from adult somatic cells reverted back to pluripotency by the forced expression of transcription factors expressed in early development (Takahashi and Yamanaka, 2006). The characteristics of these cells make them highly desirable for the utilization in regenerative therapy for PD, but also for disease modeling studies on monogenic PD forms (Li et al., 2018). Novel strategies for obtaining dopaminergic neurons directly from PD patient fibroblasts that maintain their aging and epigenetic signature, now offers the possibility to also study the idiopathic form of the disease (Drouin-Ouellet et al., 2017b; Mertens et al., 2018).

### Deriving mDAs From PSCs

Motivated by the immense potential of PSCs for use in regenerative medicine, and disease modeling studies for PD, several attempts to obtain stem-cell derived dopaminergic neurons in the dish have been made in the last years. Early work in the field relied on the incubation of stem cells with morphogenes known to be involved in dopaminergic neuron development, i.e., fibroblast growth factor 8 (FGF8), and sonic hedgehog (SHH), or co-culture with mouse stromal feeder cells, and/or midbrain astrocytes (Zhang et al., 2001; Perrier et al., 2004; Yan et al., 2005; Roy et al., 2006). These early protocols succeeded in generating tyrosine hydroxylase (TH) expressing neurons *in vitro*, albeit at low efficiency and of uncertain mesencephalic identity. However, these early differentiation attempts were limited by the then-current knowledge on mDA neuron development and mDA molecular identifiers. Indeed, the discovery that mDA neurons originated from radial glia-like cells in the floor-plate of the developing neural tube (Ono et al., 2007; Bonilla et al., 2008) changed the approaches toward the obtention of dopaminergic neurons from stem cells. It became evident that for generating *bona-fide* mDA neurons, proper induction of the floor-plate identity had to be achieved (Fasano et al., 2010). Current patterning approaches rely on the generation of high yields of neuronal progenitors by dual Smad inhibition (Chambers et al., 2009), followed by the activation of the canonical Wnt pathway to achieve proper anterior/posterior patterning and potent activation of Sonic hedgehog (SHH) signaling to induce ventral identity (Kriks et al., 2011; Kirkeby et al., 2012; Nolbrant et al., 2017). These protocols can now yield neurons that robustly express mDA identifiers such as Foxa2, Lmx1a, En1, and Corin (Kriks et al., 2011; Kirkeby et al., 2012).

These developments have opened new avenues of possibility for regenerative therapy in PD. Original cell replacement trials attempted to use mDA tissue dissected from human embryos for transplantation into PD patients (Barker et al., 2013). While promising, these trials showed to be highly variable with certain patients not benefiting from the transplant. The difficulty to standardize cell product and the presence of different types of mDA subtypes in the tissue preparation might account for the variability seen in the treatment. PSCs now offer the chance for a more standardizable and expandable source of tissue for cell replacement therapy in PD. Clinical trials using human iPSC and ESCs are currently on the way, or on the planning (Barker et al., 2017; Parmar et al., 2020), strongly supported by robust evidence in animal models that show transplanted PSC-derived mDA neurons survive, integrate, and function *in vivo* (Kriks et al., 2011; Kirkeby et al., 2012, 2017; Grealish et al., 2014; Kikuchi et al., 2017; Niclis et al., 2017; Cardoso et al., 2018; Adler et al., 2019).

With current mDA pattering approaches and iPSC technology it is now also possible to undertake patient-specific disease mechanistic and drug screening investigations for PD. Fibroblasts or peripheral blood mononuclear cells (PBMCs) obtained from PD patients can be reverted back to pluripotency, and then differentiated into mDA neurons allowing to study cellular mechanisms related to the pathology, vulnerability, and degeneration of these neurons (Li et al., 2018). This model is particularly advantageous for studying familial forms of the disease. Fibroblasts obtained from patients harboring mutations in specific genes (i.e., Snca, Lrrk2, Park2, Pink1) may be studied in comparison with an isogenic form, where the identified mutation has been reverted to wild-type by means of gene editing technologies such as Crispr-Cas9, or zinc-finger nuclease (Soldner et al., 2011). These studies can now offer new insight into distinct mechanisms associated to disease development, and neuronal degeneration, such as mitochondrial dysfunction, oxidative stress, alpha-synuclein pathology, autophagy, and neuroinflammatory responses (Li et al., 2018).

### Direct Conversion of Fibroblasts to mDA Neurons

Direct conversion is another novel and promising approach to generate mDA neurons in the dish, where the intermediate pluripotent stage is bypassed via direct conversion of fibroblasts to neuronal cells. By forced expression of only three neuronal specific transcription factors (Ascl1, Brn2, Myt1L) mice neonatal fibroblasts have been shown to convert into neurons *in vitro* (Vierbuchen et al., 2010). Other approaches for direct conversion based on overexpression of neuronal miRNAs (Victor et al., 2014) or utilization of small molecules (Hu et al., 2015) have also been developed. By adding subtype determinants to the reprogramming cocktail, fibroblasts can also be reprogrammed to specific type of neurons (Son et al., 2011; Liu et al., 2013; Victor et al., 2014; Xu et al., 2016). In particular adult mice and human neonatal dermal and lung fibroblasts have been shown to reprogram to TH-expressing neurons in the dish (Caiazzo et al., 2011; Pfisterer et al., 2011; Jiang et al., 2015).

Direct conversion now allows for the investigation of sporadic forms of neurological disorders (Drouin-Ouellet et al., 2017b). The great advantage of this methodology is that converted neurons have been shown to retain aging signatures when compared to neurons obtained via the intermediate pluripotent stage (Mertens et al., 2015; Huh et al., 2016; Tang et al., 2017). This method is therefore particularly suited for investigating neurological disorders associated with aging. By utilizing fibroblasts obtained from sporadic PD patients and directly converting them to age-matched mDA neurons, there is now the possibility to investigate the pathological processes that are exclusive to aged dopaminergic neurons.

However, it still remains challenging to obtain high yields of induced human dopaminergic neurons in the dish from dermal fibroblasts (Caiazzo et al., 2011; Pfisterer et al., 2011). Considering this strategy directly generates post-mitotic neurons that bypass any proliferative stage during the conversion process, the number of induced neurons (iNs) obtained in culture is dependent on both the expandability of the donor cell prior to conversion, and the conversion efficiency itself (the percentage of fibroblasts that successfully convert into iNs in the dish). Breakthroughs and developments focusing on increasing the efficiency of the direct conversion process from adult dermal fibroblasts and maturation of the iNs (Drouin-Ouellet et al., 2017a; Birtele et al., 2019; Li et al., 2019) will therefore be critical for both disease modeling studies and personalized diagnostics for PD.

Moreover, the molecular heterogeneity and authenticity of the obtained iNs cultures need to be characterized in detail. Single cell molecular profiling will allow for the validation of the obtained iNs by direct comparison to *bona-fide* human neurons, revealing how much of the fibroblast/neuronal signature is retained/acquired. This strategy can also be applied to elucidate on the composition and heterogeneity of the obtained iNs in culture.

### Perspectives in Cell Therapy and Disease Modeling for PD

Despite the remarkable achievements in the generation of authentic mDA neurons from PSCs, guiding the differentiation process toward subtype-specific mDA neurons still remains a challenge. Preclinical observations in animal models transplanted with human ESC-derived mDA neurons, have demonstrated the presence of both A9 (SNc) and A10 (VTA) populations in the graft as determined by the expression of TH/Girk-2 and TH/Calbindin (Grealish et al., 2014). Moreover, when these cells are placed in their homotopic location in the midbrain, graft-derived TH^+^ axonal projections can be detected in both A9 and A10 forebrain target regions (Grealish et al., 2014; Cardoso et al., 2018). Studies utilizing fetal-derived mDA neurons have also long stipulated the necessity for dopaminergic reinnervation of dorsal regions of the caudate/putamen for proper motor recovery in rodent models of PD (Bjorklund et al., 1980) and therapeutic relief in PD patients (Li et al., 2016). In agreement with these observations, the presence of the A9 component in the transplants is crucial for graft function and for reverting rotational asymmetries and motor impairment in the mouse unilateral 6-OHDA model (Grealish et al., 2010). Therefore, it is predictable that refining PSC differentiation strategies toward A9 mDA neurons would generate cell products with improved capacity for caudate/putamen reinnervation, focal dopamine release and, symptomatic relief upon transplantation into PD patients.

Several other strategies are also being proposed to improve the therapeutic outcome of cell therapy. PSC-derived mDA neurons may be modified in order to optimize axonal outgrowth and guiding, as well as neuronal activity *in vivo*. For instance, overexpression of PSA-NCAM has been shown to lead to improved fiber outgrowth of grafted mDA neurons (Battista et al., 2014). Recent studies are also unraveling the guidance machinery involved in the development of the nigrostriatal pathway (Li et al., 2014; Chabrat et al., 2017). Manipulating A9 guidance receptors, either pharmacologically or genetically, may thus improve the caudate/putamen connectivity of PSC-derived mDA neurons when transplanted into the brain. Advancements in opto- and chemogenetics also offer the possibility to remotely influence the function of the transplant (Steinbeck et al., 2015; Aldrin-Kirk et al., 2016; Chen et al., 2016). Expressing light-sensitive and designer drug responsive channels in PSCs and under specific promoters, may potentially allow for the activation or silencing of desired/undesired neurons in the graft. For these strategies to be valid, however, it is crucial that these transgenes be expressed only on functionally relevant subtypes of grafted neurons. Unveiling the molecular profile of subtype-specific mDA neurons will therefore allow for the genetic targeting of these different populations.

It will also be important that, moving forward, the cellular product obtained via differentiation of PSCs gets characterized in detail. A major concern when dealing with PSC are the possible presence of undifferentiated and dividing cells with teratoma-forming potential in the cell preparation (Brederlau et al., 2006; Roy et al., 2006; Sonntag et al., 2007), and the presence of serotonergic neurons that have been associated to graft-induced dyskinesias upon transplantation (Politis et al., 2010). Quality control assessment of PSC-derived mDA neurons are often based on immunocytochemistry and expression analysis of a selected panel of genes via qRT-PCR. However, these approaches are limited to the selection of antibodies and primers utilized in the analysis. Single cell transcriptomics can now be used in an unbiased manner to inform about the cell product composition and provide in-depth information on the presence and proportion of different cell types. In turn, this information can be used as a readout for the improvement and fine-tuning of differentiation protocols, with the aim to exclude undesired populations in the cell product and possibly enrich for specific subtypes of mDA neurons. Alternatively, the potential identification of cell surface markers specific for mDA neurons or subtypes, might allow for purification of the PSC-derived mDAs via FACS (Sundberg et al., 2013; Doi et al., 2014; Samata et al., 2016) or MACS sorting (Lehnen et al., 2017). Finally, comparing the molecular signatures of PSC-derived mDA neurons to their “gold standard” ventral midbrain fetal counterpart will elucidate the authenticity, the homogeneity and the stage of maturation of mDA cells obtained in the dish, informing for an optimal regimen for transplantation.

Refining PSCs *in vitro* patterning and direct conversion strategies toward specific mDA subtypes would also have a significant impact in disease mechanistic studies. The distinct vulnerability profile of SNc vs. VTA neurons has been well described, with the former being more susceptible to degeneration in PD (Hirsch et al., 1988; Kordower et al., 2013). Characteristics such as axonal arborization, intracellular calcium levels and mitochondrial oxidative stress have been associated to elevated cell death and Lewy body pathology in A9 neurons (Surmeier et al., 2017). It is therefore expectable that different subtypes of PSC-derived mDAs will have distinct phenotypical responses to PD-related toxic stimuli and genetic mutations *in vitro*.

Generating subtype-specific mDA neurons from PD patient iPSCs, would allow to compare resistant vs. susceptible mDA neurons, offering invaluable insight into the cellular processes behind these distinct profiles. In turn, this would enable a more efficient and tailored approach toward drug screening and the identification of novel therapeutic targets.

Recent developments in 3D culturing and neuronal differentiation have also led to the development of protocols to generate midbrain organoids from PSCs and neural stem cells (Jo et al., 2016; Monzel et al., 2017). These structures maintain a certain level of spatial organization, establish complex neuronal networks and give rise to distinct cell types, including glial cells, which allow for another level of disease mechanistic and modeling investigations for neurodegenerative diseases (Kim et al., 2019; Smits et al., 2019). Moreover, these organoids have the advantage to recapitulate some aspects of VM development and to contain both A9 and A10 mDA neurons (Jo et al., 2016; Monzel et al., 2017). This model therefore holds the potential for unprecedented investigations on the role of cell-cell and cell-matrix interactions on subtype-specific susceptibility to PD. However, oxygen and nutrient deficiencies in the core of larger organoids (Mansour et al., 2018), are hurdles to overcome toward the obtention of fully self-organized VM-like structures.

**FIGURE 1:**
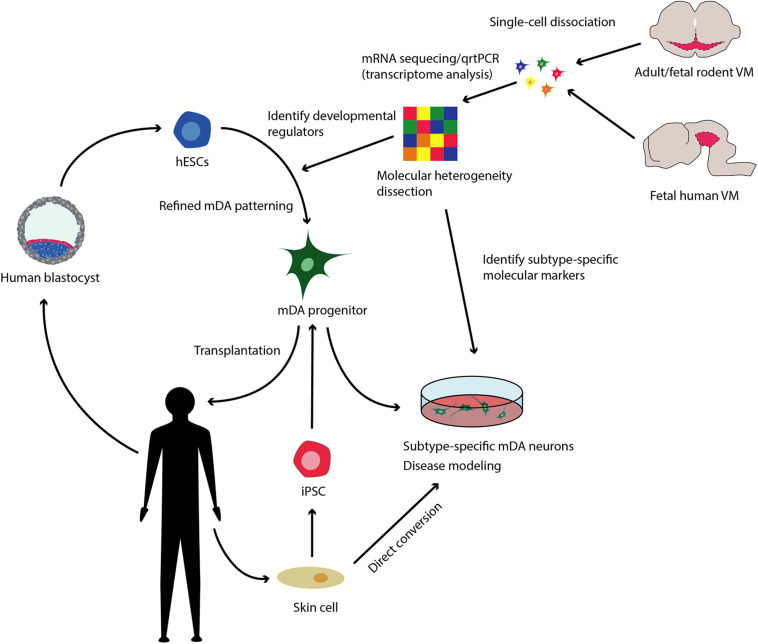
Refining mDA patterning and direct conversion toward subtype-specificity. Human embryonic stem cells (hESCs) derived from the inner mass of the blastocyst and induced pluripotent stem cells (iPSC) obtained from reprogrammed somatic cells, can be patterned to midbrain dopaminergic neurons (mDA) *in vitro*. These cells can be utilized as both a source of transplantable tissue for the treatment of Parkinson’s disease (PD) and for modeling PD in the dish allowing for mechanistic and drug discovery studies. Reprogramming skin cells from both healthy and diseased donors directly to mDAs, also constitutes a promising approach toward disease modeling studies. Single cell transcriptomic analysis now allow for the dissection of the molecular diversity of adult mDAs and of the molecular orchestrations behind mDA development. Determining key molecular regulators of mDA development could lead to refined protocols to pattern hESCs and mDAs toward subtype specificity. Identifying subtype-specific molecular markers, will allow for quality control monitorization of the cell product for the presence of the desired mDA subtypes *in vitro*.

In short, there is a need for a more detailed understanding of mDA development and diversity in adults. Identifying molecular determinants of mDA differentiation, specification and maturation as well as markers defining mDA neurons diversity would permit the optimization of stem cell patterning and direct conversion approaches toward subtype-specificity, in turn providing a framework for quality control assessment of cell product authenticity and composition (see Figure 1).

## Investigating mDA Neuron Development and Diversity at the Single Cell Level

Dopaminergic neurons express several enzymes and proteins involved in the synthesis (TH; DOPA decarboxylase), vesicular packaging (Vmat2) and synaptic reuptake (Dat) of dopamine. At the ventral mesencephalic level, the specification, differentiation and maintenance of this phenotype is orchestrated by the expression of early (Lmx1a/b, Foxa1/2, En1/2) and post-mitotic (Nurr1 and Pitx3) transcription factors (reviewed in Arenas et al., 2015). The traditional classification of the dopaminergic neurons of the ventral midbrain divides this region into two major compartments: the substantia nigra pars compacta and the ventral tegmental area. At the molecular level, Girk2 and Calbindin have been traditionally utilized to differentiate between these two structures, respectively, but mDA neurons expressing these markers are not mutually exclusive and can be found expressed in both compartments, albeit at different levels, in both humans and mice (Reyes et al., 2012). However, this simplistic dual classification of the mDA system fails to truly represent the versatility and complexity of behavioral functions and neurological disorders associated to this brain region.

Over the years, several efforts have been made to further resolve the heterogeneity of the ventral mesencephalon based on features such as cellular morphology, electrophysiological properties, axonal projections, vulnerability and behavioral function (Bjorklund and Dunnett, 2007a; Roeper, 2013; Yetnikoff et al., 2014). Moreover, several attempts to compartmentalize the ventral midbrain at the molecular level have described a myriad of region-specific associated markers (reviewed in Anderegg et al., 2015). These studies have been based on the characterization of the subtype- and temporal-specific expression of a limited number of genes by protein and mRNA detection in histological slices (Smits et al., 2013) and gain and loss of function studies in transgenic animals (Panman et al., 2014). While providing important insight into mDA neuron development and heterogeneity, these studies are limited by the number of markers that can be interrogated at the same time. Molecular profiling by analysis of multiple genes at the bulk level – average across all cells in SNc vs. VTA – has also been described (Chung et al., 2005; Greene et al., 2005). However, these studies do not provide an anatomically unbiased picture of the cellular heterogeneity in the mDA neurons and may therefore overlook the presence of previously unidentified neuronal subtypes.

Recent developments in single cell technology allow the molecular profiling of the central nervous system at unprecedented level (Tang et al., 2009; Tasic et al., 2016; Zeisel et al., 2018). In light of this, several reports have looked at the gene expression patterns at different stages of mDA neuron development in both human and rodent tissue at the single cell level. These studies provide unique insight into the diversity of the adult ventral midbrain, the molecular orchestrations behind mDA neurons development and the genetics associated to sporadic PD (Poulin et al., 2014; La Manno et al., 2016; Kee et al., 2017; Hook et al., 2018; Tiklová et al., 2019; see Table 1). The methodological pipeline behind these investigation consist on the dissociation and isolation of mDA tissue to the single cell level by microfluidics or FACS sorting – in turn allowing for the enrichment of specific cell populations based on reporter gene expression under specific mDA promoters -, the generation of cDNA libraries from individual cells, decoding of the transcriptome by next generation sequencing or multiplexed qRT-PCR, and bioinformatic analysis of the data sets (Stegle et al., 2015; Chen et al., 2019). *Post hoc* analysis at the histological level, by immunohistochemistry, in-situ hybridization, and spatial transcriptomics can then be utilized to locate at the cytoarchitectonic level the newly identified mDA neuron subtypes.

**TABLE 1:** Summary of single-cell studies on mDA development and diversity.

**References**	**Species**	**Dev stage/Age**	**Selection**	**Nr cells sampled**	**Transcriptional analysis**	**Highlighted findings**
Tiklová et al., 2019	Mice	E13.5; E15.5; E18.5 P1; P7; P90	Pitx3-EGFP	1106	RNA-seq	Two main group of mDAs based on the level of expression of Dat (Dat-high and Dat-low); further sub-classification into seven distinct mDA subtypes; description of trajectory-specific transcription factors during mDA development.
Hook et al., 2018	Mice	E15 P7	Th-EGFP	396	RNA-seq	Presence of postnatal (P7) neuroblast population in dorsal SNc.
La Manno et al., 2016	Human	w6; w7; w8; w9; w10; w11	–	1977	RNA-seq	Description of diversity of human radial glia cell types; identification of three prenatal and five postnatal mouse mDA subtypes; machine learning approach to assess quality of hESC-derived mDAs.
	Mice	E11.5; E12.5; E13.5; E14.5; E15.5; E18.5; P19-27	Wild type	1907		
		P28; P56	Dat1-Cre/tdTomato	245		
Kee et al., 2017	Mice	E10.5; E11.5; E12.5; E13.5	Lmx1a-EGFP	550 (+ and – fraction)	RNA-seq	Close transcritptional relationship between mDAs and STN lineages; identification of early markers that predict graft outcome (see also Kirkeby et al., 2017).
Poulin et al., 2014	Mice	P4	Dat1-Cre/tdTomato	159	qRT-PCR	Identification of six neuronal mDA subtypes in mouse.

These studies are now providing an invaluable framework toward generating mDA neuron subtypes *in vitro*. The identification of molecular markers for distinct mDA populations, opens the door for targeted interrogation at the functional level, in turn identifying potential populations of interest for *in vitro* engineering. Moreover, resolving the molecular orchestrations behind mDA neurons development may allow for the identification of early fate determinants that can be utilized to optimize differentiation of PSCs to mDA neurons. Finally, single cell technology may be directly applied to profile the engineered mDA neurons for authenticity, maturity and heterogeneity.

### Identifying Function and Molecular Profile of mDA Neuron Subtypes

**FIGURE 2:**
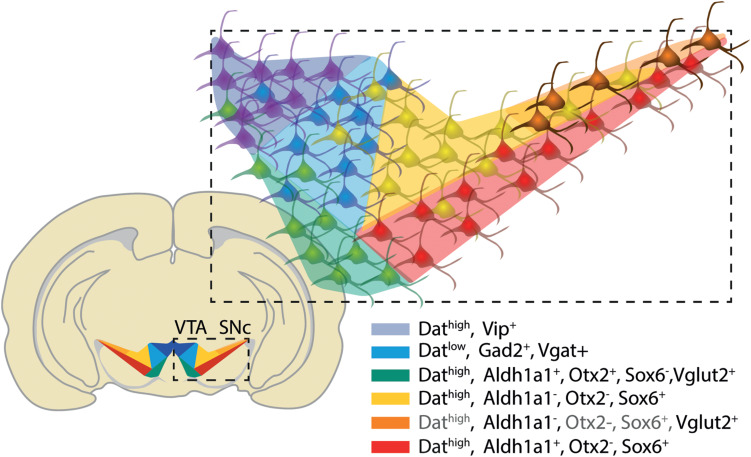
Anatomical compartmentalization and molecular profile of dopaminergic neurons within the ventral mesencephalon of the adult mouse brain. The combinatorial expression of Sox6, Otx2, and Aldh1a1 appears to segregate the dorsal tier and ventral tier of SNc, and lateral VTA (Poulin et al., 2014; La Manno et al., 2016). Neurons expressing low levels of Dat (Tiklová et al., 2019) and genes associated with GABAergic (Vgat; Gad2) and glutamatergic machinery (Vglut2) possibly identify neurons co-releasing both dopamine and GABA or glutamate, respectively. An additional population of Vglut2 expressing mDA neurons has been identified in substantia nigra pars lateralis (Poulin et al., 2018). The expression of Vip is exclusive to mDA neurons in the dorsal VTA and periaqueductal gray/dorsal raphe. Gray font identify genes with dubious expression in the region.

Up to seven molecularly distinct dopaminergic neuronal subtypes have been identified in the adult rodent mDA neurons in the advent of single cell profiling studies (Poulin et al., 2014; La Manno et al., 2016; Tiklová et al., 2019; see Figure 2). These populations can be mapped within the SNc, VTA and periaqueductal gray and share the expression of canonical floor plate (Otx2, Corin1, Wnt1, Lmx1a, and Foxa2; La Manno et al., 2016) and mDA neuron markers (Foxa1, En1/2, Lmx1b, Pitx3, and Nr4a2; Poulin et al., 2014). While these transcription factors are commonly utilized to confirm the mDA identity of stem cell derived products (Kriks et al., 2011; Kirkeby et al., 2012) they do not, however, distinguish between distinct mDA subtypes.

The description of the molecular profile of these neuronal subtypes at the single cell level now provides unique genetic entry points for the dissection of their connectome and function (Poulin et al., 2018). The identification of mDA subpopulations that elicit selective vulnerability and degeneration in PD, and involvement in motor control, would pose as an interesting candidate for *in vitro* engineering with cell therapy and disease modeling for PD in mind. These reports have consistently highlighted Aldh1a1 as defining a subset of mDA neurons at the ventral tier region of the SNc (Poulin et al., 2014; La Manno et al., 2016; Tiklová et al., 2019; see Figure 2). Aldh1a1 expressing mDA neurons have been previously described in the rodent and human SNc (McCaffery and Drager, 1994; Galter et al., 2003). This population has been shown to be vulnerable in PD, to innervate dorsolateral regions of the caudate putamen and to be involved in motor skill learning and control (Liu et al., 2014; Sgobio et al., 2017; Poulin et al., 2018; Wu et al., 2019). While Aldh1a1 expressing neurons are also present in ventromedial VTA, single cell profiling shows that the combined expression with Sox6 seems to be sufficient to define the SNc subtype (Poulin et al., 2014; La Manno et al., 2016; see Figure 2). However, further analysis needs to be done to assert the singularity and function of the identified subtypes of mDA neurons; these reports prove to be resourceful for moving toward the identification of mDA neuron subtypes that involve distinct functions in the brain and associate to distinct CNS disorders. To describe the unique molecular profile of these subtypes will also provide a readout for monitoring the differentiation of PSC-derived mDA neurons via immunocytochemistry or qRT-PCR.

### Refining mDA Neuron Patterning

The analysis of the gene expression dynamics at the single cell level and at early embryonic stages can reveal distinct populations in adults that share a common early developmental trajectory, while distinguishing these closely related lineages at the molecular level. By sequencing single cells of E10.5-E13.5 Lmx1a-EGFP mice, Kee and colleagues identified two main axis of development stemming from Lmx1a neuronal progenitors that shared the expression of commonly utilized mDA cells identifiers such as Lmx1a/b, Foxa1/2, Otx2, Foxp1/2, and Msx1 (Kee et al., 2017). While one axis represented the subthalamic neurons (STN) lineage and the other the dopaminergic lineage, reveal that these populations are closely related at early developmental stages. The authors further identified sets of genes that can differentiate between the dopaminergic (En1 and Pitx3) and the STN lineage (Barhl1, Pitx2 and Epha3). The use of this combination of transcription factors as predictive markers, Kirkeby and colleagues described an improved PSC differentiation protocol that enrich for mDA cells and exclude STN progenitors from the cell product (Kirkeby et al., 2017).

These early development profiling studies can also inform on the timing and molecular orchestrations behind mDA subtype specification and segregation. LaManno and colleagues used this approach to sequence single cells from human (w6-11) and mouse (E11.5-P21) ventral midbrain. They were able to identify early emerging diversity in the ventricular zone already at the radial glia stage. Interestingly, the authors identified only three clusters of embryonic post-mitotic dopamine neurons (DA0, DA1, and DA2) but were able to distinguish five dopaminergic neuronal populations (DA-SNC, DA-VTA 1-4) in adult mice, suggesting that adult dopaminergic subtypes only emerge postnatally and may be the result of environmental cues rather than early programming events (La Manno et al., 2016). Identifying the agents at play behind mDA post-mitotic differentiation would prove pivotal toward guiding induced mDA neurons toward desired subtypes.

Finally, these reports provide online resources containing detailed information on individual gene expression patterns. The information therein provides a blueprint for identifying transcription factors associated to early patterning and subtype specification, which can be utilized to guide stem cell differentiation and direct conversion strategies toward subtype specificity.

### Direct Profiling of Induced mDA Neurons

Single cell transcriptomics can also be applied to profile directly induced and PSC-derived mDA neurons. By comparing gene expression patterns of induced mDA neurons to their *bona fide* human fetal counterpart, information on the composition of the cell product as well as fidelity and maturation stage of the differentiated cells can be inferred. By analysing the composition and the transcriptomic profile of single cells obtained from hESCs and hiPSCs-derived DA neuron cultures at distinct timepoints (from days 0 to 63), LaManno and colleagues were able to identify 14 distinct cell types that resembled to the 25 identified populations in the human fetal VM tissue (La Manno et al., 2016). The authors also describe a machine learning tool to quality control assessment and scoring of individual PSC-derived mDA neurons in comparison to their fetal counterpart, based on gene expression profiles. Such approach could be used to identify new genes involved in mDA neuron development and to improve differentiation protocols.

Alternatively, PSC-derived mDA neurons may also be profiled after a period of maturation *in vivo*. Comparing gene expression patterns before and after transplantation in animal models, may inform on the composition of the transplants and the effect of environment on mDA neurons maturation.

Finally, single-cell sequencing mDA neurons induced from PD patient-derived iPSCs, may also reveal gene regulatory features associated with the development of the disease, by exploiting the cellular heterogeneity and distinct pseudotemporal disease states of individual mDA neurons (Lang et al., 2019). Further studies utilizing novel single cell sequencing methods are required for a full comprehension on the subtypes and fidelity of directly induced or PSC-derived mDA neurons, as well as molecular features of PD.

## Concluding Remarks

Recent technical achievements in single cell transcriptomics are now providing remarkable insight into the diversity and development of the dopaminergic neurons of the ventral mesencephalon. However, certain technical caveats related to these studies have to be taken into consideration. The current approaches herein discussed, rely on the pre-selection of mDA neurons based on the expression of reporter genes driven by specific dopaminergic promoters (Dat, Lmx1a, Pitx3). This cellular enrichment approach allows for more cells of interest to be profiled, providing a more comprehensive and in-depth analysis of the mDA neurons diversity. However, this strategy might lead to certain subtypes of mDA neurons being overlooked, as they may express low levels of the selection gene. Indeed the description of mDA neurons that express a low level of Dat – that may account for TH/GABA co-releasing neurons or non-TH producing neurons in the VTA (Tiklová et al., 2019), may have been overlooked by previous reports basing their selection step on the expression of Dat (Poulin et al., 2014; La Manno et al., 2016).

In addition, some specific subtype of neurons might be more vulnerable to single cell dissociation, and therefore may be underrepresented in the analysis. Several evidence also indicate that a large number of mRNAs are transported and translated in the axon terminals (Shigeoka et al., 2016). These mRNAs are not detected in dissociated neurons, likely raising the probable omission of key regulators of axon outgrowth and guidance. Approaches that can select for mDA neurons early at the developmental stage, complemented with spatial transcriptomic at the tissue level (Stahl et al., 2016) and the inclusion of mDA terminals in the analysis (Sanz et al., 2009), will provide a more complete picture of the diversity and development of the ventral mesencephalon.

Furthermore, despite these remarkable advances in mDA genetic profiling and molecular diversity, no significant breakthroughs in the generation of subtype-specific mDA neurons has emerged to date. Considering the limitations associated to the access of human fetal tissue, it remains to be ascertained if current single cell sequencing technologies are sensitive enough to identify and profile the full range of mDA progenitors in the developing ventral midbrain.

Moreover, early specification events may be insufficient to commit mDA progenitors to become subtype specific mDA neurons in the adult. The role of the cell extrinsic factors, following the migration of postmitotic mDA neuroblasts within the VM, may influence their terminal differentiation toward distinct mDA subtypes. For instance, laminins (extracellular matrix proteins) have been shown to promote mDA cells differentiation *in vivo* (Zhang et al., 2017) and to improve PSC patterning to mDA neurons *in vitro* (Doi et al., 2014; Kirkeby et al., 2017; Ahmed et al., 2019). Moreover, early dopaminergic signaling may also influence mDA neuron development (Kim et al., 2006) and PSC differentiation (Bono et al., 2018). While none of these studies investigated the acquisition of subtype specificity, they highlight the potential role of cell-matrix and cell-to-cell interactions in influencing mDA neuron development and differentiation. Further investigations on early synaptic connectivity and matrix/glia interactions may further elucidate the mechanisms and players involved in mDA neuron subtype acquisition. Utilizing VM-patterned organoids as an *in vitro* model of mDA development may now offer invaluable insight into these processes. For instance, single cell sequencing methods could be applied to dissect the developmental features of mDA neurons in ventral midbrain-patterned organoids, as already described in cortical organoid models (Amiri et al., 2018; Velasco et al., 2019).

Finally, variations in the genetic background, subclonal mutations and passage number (Volpato and Webber, 2020), affect the differentiation potential of different PSCs lines and should be taken into consideration for their potential to generate distinct subtypes of mDA neurons.

In short, translating mDA neuron diversity and developmental findings to stem cell differentiation and lineage conversion methodologies, would greatly impact regenerative therapy approaches and disease modeling studies for Parkinson’s disease.

## Author Contributions

TC wrote the manuscript. ML revised the manuscript.

## Conflict of Interest

The authors declare that the research was conducted in the absence of any commercial or financial relationships that could be construed as a potential conflict of interest.
